# Treatment of Internal Hemorrhoids by Hypodermic Medication

**Published:** 1879-12-20

**Authors:** Geo. Ross

**Affiliations:** Richmond, Va., Special Lecturer on Diseases of Women, Medical College of Virginia


### *TREATMENT OF INTERNAL HEMORRHOIDS BY HYPODERMIC MEDICATION.*

BY GEO. ROSS, M.D., RICHMOND, VA.,

Special Lecturer on Diseases of Women, Medical College of Virginia.

Dr. Edmund Andrews, of Chicago, in the July number of the Philadelphia Monthly Review, contributed a most valuable article on the cure of hemorrhoids by hypodermic injection. And as any clinical experience tending to confirm the verity of the suggestions in that paper strengthen it, and may lead others to their adoption, I desire to add one to the list of recorded successful cases.

Miss B. has been a sufferer from internal bleeding hemorrhoids for nearly two years, at times enduring such agony as to contemplate suicide. She could not stand or walk without the protrusion of one or more tumors through the sphineter ani, and after stool, besides the painful and disagreeable necessity of replacing the everted rectum and mass of tumors, was obliged to lie down for a considerable length of time before she was equal to the demands of her vocation. She had suffered so long and acutely, and every agent which her medical adviser had suggested having so signally failed in affording the slightest relief, that in sheer desperation she took refuge in opium, chloral, and whiskey, unconsciously using such quantities that she became a prey to their power. Some of her friends thought her verging on lunacy.

Once arrangements were made for a surgical operation, and she was actually on the table prepared for the ligation of the tumors, but for some reason it was postponed. Of ointments and lotions she had used endless quantities, and losing faith in all medical men, had relapsed into a stolid indifference, preferring to endure the "ills she had, rather than fly to those she wot not of." About the last of September an attack of inflamed hemorrhoids made her so desperate she consulted me. Upon examination, I decided at once to adopt the hypodermic method of medication, and to employ the weakest solution of carbolic acid advised in the paper referred to. The bowels having been previously thoroughly emptied by enema, on the morning of the 4th of October I injected ten drops of carbolized oil (ʒix olive oil, and ʒi cryst carbolic acid) into the centre of *one* tumor, throwing the fluid in very slowly, and retaining the needle in situ until it could be thoroughly distributed through the tissues. The almost instant effect was the whitening and partial corrugation of the mucous membrane. I then injected alongside ⅛ grain sulph. morphia, replaced all the tumors above the internal sphincter muscle, and made my patient comfortable in bed. The operation was not very painful, but the pain increased considerably in two hours, apparently because of the incessant action of the "sphincter," and lasted an hour or two. To relieve this, as well as to assist in the absorption of the tumors, I directed the application, night and morning, of this ointment:

| | | |
|---|---|---|
| ℞ | Iodoform | ʒ i |
| | Acid carbolic | grs. xv |
| | Acid Tannic | grs. xv |
| | Ext. belladon | grs. viii |
| | Pulv. opii | grs. viii |
| | Vaseline | ʒ i |

To guarantee a semi-solid stool, she took each morning, immediately before breakfast, a small wineglassful of Hunyadi Janos water. On the 11th of October I repeated the treatment, injecting *two* tumors, and omitting the morphine. That tumor originally injected was much diminished in size. On the 18th she was menstruating, so I was forced to postpone my injection until the 25th. At that time I could only find two tumors, and they so small that I should not have thought it necessary to do anything for them except upon the ground of making "assurance doubly sure." She declared herself perfectly well, so far as the piles were concerned, and said that she almost wondered whether she could really be the same individual. The third and last operation was almost painless—she did not lose a day from her school—was equal to any amount of exercise during treatment, a thing absolutely impossible before, because as soon as a tumor escaped through the "sphincter," a most intolerable strangury came on. Hence, I conclude that except in cases where the hypodermic method fails, I shall discard the ligature and ecraseur in the management of "internal hemorrhoids."—*Southern Clinic.*

---

**Means of Arresting the Epileptic Attack.**—At the *Societie de Biologie* on the 5th of July, M. Brown Sequard said that he had learnt from a negro, that an attack of epilepsy may be arrested by pulling the great toe. Moreover, he had himself verified the correctness of the fact upon twenty-one patients.—*Hospital Gazette.*

